# Correlation Structure in Micro-ECoG Recordings is Described by Spatially Coherent Components

**DOI:** 10.1371/journal.pcbi.1006769

**Published:** 2019-02-11

**Authors:** Nicholas Rogers, John Hermiz, Mehran Ganji, Erik Kaestner, Kıvılcım Kılıç, Lorraine Hossain, Martin Thunemann, Daniel R. Cleary, Bob S. Carter, David Barba, Anna Devor, Eric Halgren, Shadi A. Dayeh, Vikash Gilja

**Affiliations:** 1 Physics, University of California San Diego, La Jolla, CA, United States of America; 2 Electrical and Computer Engineering, University of California San Diego, La Jolla, CA, United States of America; 3 Department of Neurosciences, University of California San Diego, La Jolla, CA, United States of America; 4 Materials Science and Engineering, NanoEngineering University of California San Diego, La Jolla, CA, United States of America; 5 Department of Radiology, University of California San Diego, La Jolla, CA, United States of America; 6 Department of Neurosurgery, University of California San Diego, La Jolla, CA, United States of America; 7 Martinos Center for Biomedical Imaging, Massachusetts General Hospital, Harvard Medical School, Charlestown, MA, United States of America; Harvard University, UNITED STATES

## Abstract

Electrocorticography (ECoG) is becoming more prevalent due to improvements in fabrication and recording technology as well as its ease of implantation compared to intracortical electrophysiology, larger cortical coverage, and potential advantages for use in long term chronic implantation. Given the flexibility in the design of ECoG grids, which is only increasing, it remains an open question what geometry of the electrodes is optimal for an application. Conductive polymer, PEDOT:PSS, coated microelectrodes have an advantage that they can be made very small without losing low impedance. This makes them suitable for evaluating the required granularity of ECoG recording in humans and experimental animals. We used two-dimensional (2D) micro-ECoG grids to record intra-operatively in humans and during acute implantations in mouse with separation distance between neighboring electrodes (i.e., pitch) of 0.4 mm and 0.2/0.25 mm respectively. To assess the spatial properties of the signals, we used the average correlation between electrodes as a function of the pitch. In agreement with prior studies, we find a strong frequency dependence in the spatial scale of correlation. By applying independent component analysis (ICA), we find that the spatial pattern of correlation is largely due to contributions from multiple spatially extended, time-locked sources present at any given time. Our analysis indicates the presence of spatially structured activity down to the sub-millimeter spatial scale in ECoG despite the effects of volume conduction, justifying the use of dense micro-ECoG grids.

## Author summary

Electrocorticography (ECoG) is a type of electrophysiological monitoring that uses electrodes placed directly on the exposed surface of the brain. ECoG is a promising technique for studying the brain, and EcoG signals can be used to control brain-computer interfaces. Advances have made it possible to record simultaneously with an increasing number of smaller, and more closely spaced electrodes. However, a property of electrical recording from outside the brain is that common signals appear on different electrodes at different locations, and this affects decisions about how to best distribute a limited number of electrodes to maximize the information that can be gathered. Large spacing of electrodes around one centimeter apart on the brain’s surface has proven useful for clinical and research use, but how much benefit there is to recording from more locations in a smaller area remains to be answered. We found that we can explain the commonality between the different locations as the combination of different patterns of brain activity that are present at multiple electrode locations, and that signals recorded from very closely spaced electrodes, around a millimeter or less apart, are able to identify patterns that are at this small scale.

## Introduction

Electrical recording from the brain surface, known as electrocorticography (ECoG), is becoming more common due to technological advances that enable recording from large cortical surface area with high temporal resolution and far better spatial resolution than non-invasive EEG [1, 2]. ECoG has also been used as an alternative to penetrating intracortical recording electrodes in brain-computer-interface (BCI) applications [3–9] due to its less invasive nature and long-term stability that are important features for driving drive BCI’s [3]. Electrodes designed for BCI will typically have more closely spaced electrodes to target specific cortical regions compared to clinical ECoG, in which large cortical coverage is important. Recently, high density ECoG grids have become more common, and many questions on the properties, uses, and design of these grids, e.g., what is the optimal spacing for the electrodes [8–11], remain to be answered.

Recording hardware sets an upper limit on the number of channels that can be simultaneously recorded. This creates a tradeoff in designing ECoG electrode grids between coverage and resolution. Clinical grids are typically on the larger coverage side, with 1 centimeter being a typical pitch between electrodes. BCI and research applications have pushed for more resolution in order to place more electrodes near cortical regions of interest [3, 6, 8–10, 12–17]. A challenge of scaling down the size of ECoG grids is that low impedance electrodes improve signal quality, but electrode impedances increase as the contact area decreases [18]. Combining fabrication techniques that allow for smaller, more closely spaced ECoG contacts with novel materials that can significantly reduce impedance makes very small contact sizes feasible. In the present study, we used electrodes coated with PEDOT:PSS on gold traces embedded in a parylene-C substrate [19, 20] with contact diameter as small as 20 microns and pitches as low as 200 microns. Hereafter, we will refer to these ECoG electrode grids as micro-ECoG. Previous work has shown that recordings with micro-ECoG electrodes are more similar to intracortical recordings than to the recordings from larger clinical ECoG electrode grids [14].

The primary signal of interest in ECoG recordings is the lower frequency component (less than ~200–500 Hz) called the local field potential or LFP. LFP is an uncertain signal in that its precise physiological and spatial origins are poorly understood [21, 22]. Much of the difficulty both in studying and using LFP is due to its lack of spatial specificity, that is, the potentials are an aggregate of nearby activity—in contrast to single- or multi-unit electrophysiological signals which are indicative of action potentials very near the recording site [23]. The spatial extent of LFP is itself an area of study [24–26] with a dependence on the geometry and activity of the region generating the signal. The spread of the potentials manifests itself in ECoG as similar signals being recorded on different electrodes. This feature of the potentials at different electrodes can be used to interpret the signals [27, 28] or guide the design of electrode grids to optimally sample the cortical surface.

To quantify the similarity between electrodes, previous studies examined the correlation or coherence of EEG, ECoG, and intracortical electrodes as a function of inter-electrode distance by averaging the correlation or coherence across many pairs separated by the same distance [8, 13–15, 28–31]. Most of these studies are in human or nonhuman primate, with some early investigations on smaller mammals, reptiles, and invertebrates. In ECoG recordings these studies have shown a consistent nearly monotonic decrease in the correlation as the electrode separation increases that exhibits a roughly exponential shape. Also consistent across the studies is a dependence of the correlation/coherence on the frequency band examined.

It is expected that the correlation/coherence would tend to zero (or bias level) at large distances, and this can be seen in EEG and clinical ECoG [14, 30, 31]. On the other hand, the correlation should approach 1 as the separation approaches 0. This is the case because the brain is a conductive medium, and for finite sources distant from the electrode in a volume conductor the potential will be the sum of all of the sources with amplitudes attenuated with distance. The distance at which the similarity will effectively approach 1 will depend on both the geometry of the sources and the properties of the medium. An example of this is discussed in [29, 32], where ECoG correlation between submillimeter-spaced electrodes is mostly close to 1 while correlation between even more closely spaced intracortical electrode pairs is frequently much lower than 1. This sub-millimeter regime in ECoG is largely unexplored, and at the smallest distances in most previous studies the correlation or coherence is significantly below 1, meaning there is still room to explore smaller electrode spacing.

On the other hand, for practical purposes recording ECoG at the scale in which the neighboring pairs measure very close to the same signal is not optimal because this would mean a large amount of redundancy between channels. The spacing should be guided by the spatial extent of features of interest. It has been suggested that contacts should be less than 5 mm apart for adequate sampling of gamma band in human ECoG [12], that for BMI applications subdural electrodes in humans be spaced 1.7 mm apart and in rat 0.6 mm apart [9], and that by halving the spacing of electrodes from 3.5 to 1.68 mm implanted in minipig, more and separate response peaks could reliably be identified [10]. The optimal separation will depend on factors such as species, location, and the nature of the activity of interest, but in general it will be difficult experimentally to recognize the optimal spatial resolution for a specific application until it is exceeded. However, we expect there may be an approximate resolution to use as a rule of thumb for each species.

We analyze the similarity of micro-ECoG with inter-electrode spacings down to 0.4 mm in human recordings and 0.2 mm in mouse. In agreement with past studies, we found that on average the signals were more similar for more closely spaced electrodes. With exceptions, higher frequency components of the signal showed a larger decrease in similarity with distance.

We also investigated the nature of the pairwise correlation between electrodes across the electrode grid. For a group of closely spaced electrodes to be correlated to each other there must be parts of their signals that are common between each channel pair, and parts that are independent to each electrode. The relative size, distribution, and properties of these signals determines the correlation between each pair of electrodes across the whole array. There are several analyses that are tailored to finding common signals across multiple channels commonly used on electrophysiological data such as principal component analysis (PCA), factor analysis, or independent component analysis (ICA). We modeled the effect of the properties of the components on the correlation structure, and then used ICA on the data to identify and separate common signals and find how they are distributed across the grid. We found that there are smoothly distributed sources present in the data, and due to the linearity of the ICA decomposition, show that the spatially coherent ICA components account for much of the correlation structure in the data.

## Results

### Distance-averaged correlation

**Fig 1:**
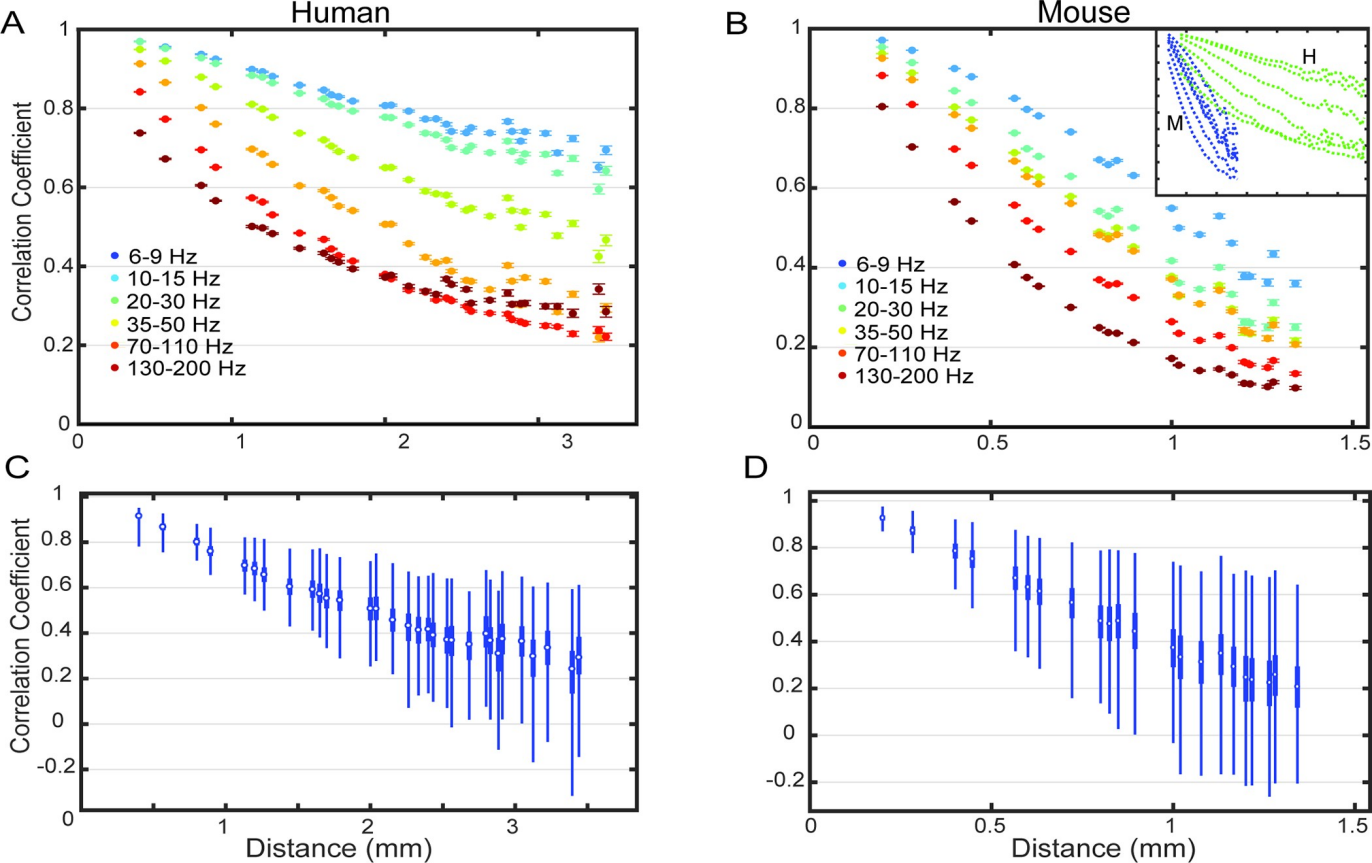
Correlation averaged equidistant electrode pairs (DAC) (A) Results for a human subject 1. Each color represents a frequency band. Error bars are 95% confidence interval of the mean across all time windows and equidistant electrode pairs. (B) DAC for mouse 1. Inset shows (A) and (B) plotted on the same scale for comparison between human (green) and mouse (blue) DAC. (C-D) The distribution across time obtained by averaging only within each window. Boxplot of the distributions of the correlation values over time for one human and one mouse subject in the 35–50 Hz band. The distributions are across all 2.0 s windows with each value the average at each distance of the correlation.

Human subjects (n = 2) were implanted with a grid of 56 electrodes with 0.4 mm center-to-center distance referenced to another larger (3 mm diameter) ECoG electrode a few centimeters away, and mice (n = 2) were implanted with 32-electrode square grids with 0.2 mm or 0.25 mm spacing with a subcutaneous reference near the skull. After removing poor channels and potential artifacts, the signals were bandpass filtered into 6 different bands, and separated into non-overlapping 2.0 second windows (527 windows for subject 1, 326 for subject 2, 1,486 for mouse 1, and 893 for mouse 2). The Pearson correlation coefficient (referred to as simply correlation) was calculated for each window between all pairs of channels on the ECoG grids for each filter. After averaging correlations across equidistant electrode pairs as in [26] which we will call distance-averaged correlation (DAC), we see that the correlation between pairs of channels decreases on average as the distance between the electrodes increases (Fig 1). The values of correlation in Fig 1 are averages of correlation calculated in 2 second segments of the data across all segments and channel pairs that share the same spacing. We find similar values to previous studies of the correlation as a function of electrode distance [14, 15, 30]. Correlation was analyzed separately for different frequency bands due to the 1/*f* nature of the signal power, and that the presence of distinct processes present in different bands are common in electrophysiology studies. Also, it is a well-known feature of ECoG that common activity in lower frequencies tends to appear over larger regions than high frequency activity. We find a similar trend in the correlation plots with some exceptions between adjacent frequency bands, and that between the two commonly used bands in electrophysiology studies, beta (15–30 Hz), and high gamma (70–110+ Hz), the difference is quite large as expected.

The differences between the results in human and mouse is also large (Fig 1B inset). In mouse the correlations fall below 0.5 within 1.5 mm while in human even the highest frequencies are correlated above 0.5 up to around 2 mm. The low frequencies in human are noticeably more correlated across distances of a few millimeters. The values within the 2.0 second time windows vary considerably but are concentrated near the mean values (Fig 1C and 1D).

### Component modeling

Correlation is a measure of to what degree two signals are similar, but an alternative approach to view the similarity is to look for commonality among all the signals simultaneously rather than an aggregate of pair-wise comparisons. There are a few methods which are commonly used in electrophysiology for identifying common signals that are present on multiple channels: principal component analysis (PCA), factor analysis, and independent component analysis (ICA). All are built around the assumption that there exists a set of signals that are present in the data with various amplitudes across all of the channels. ICA and factor analysis were developed to find underlying signals while PCA was not. Factor analysis assumes the components are drawn from a Gaussian distribution (across time samples, not channels), which does not describe the data, especially the sinusoidal signals obtained after bandpass filtering. We used ICA because we expect it to best find the underlying signals, and it is commonly used for this purpose. An important point in using ICA (as well as PCA and FA) is that the geometry of the recordings is not an input to the algorithm. The inputs are a set of time series (in this case) with no particular ordering, arrangement, or any other information relating the channels to one another. Therefore, an orderly spatial arrangement of the ICA results has been taken as an indication of the efficacy of ICA in separating sources, and is compelling in many cases.

**Fig 2:**
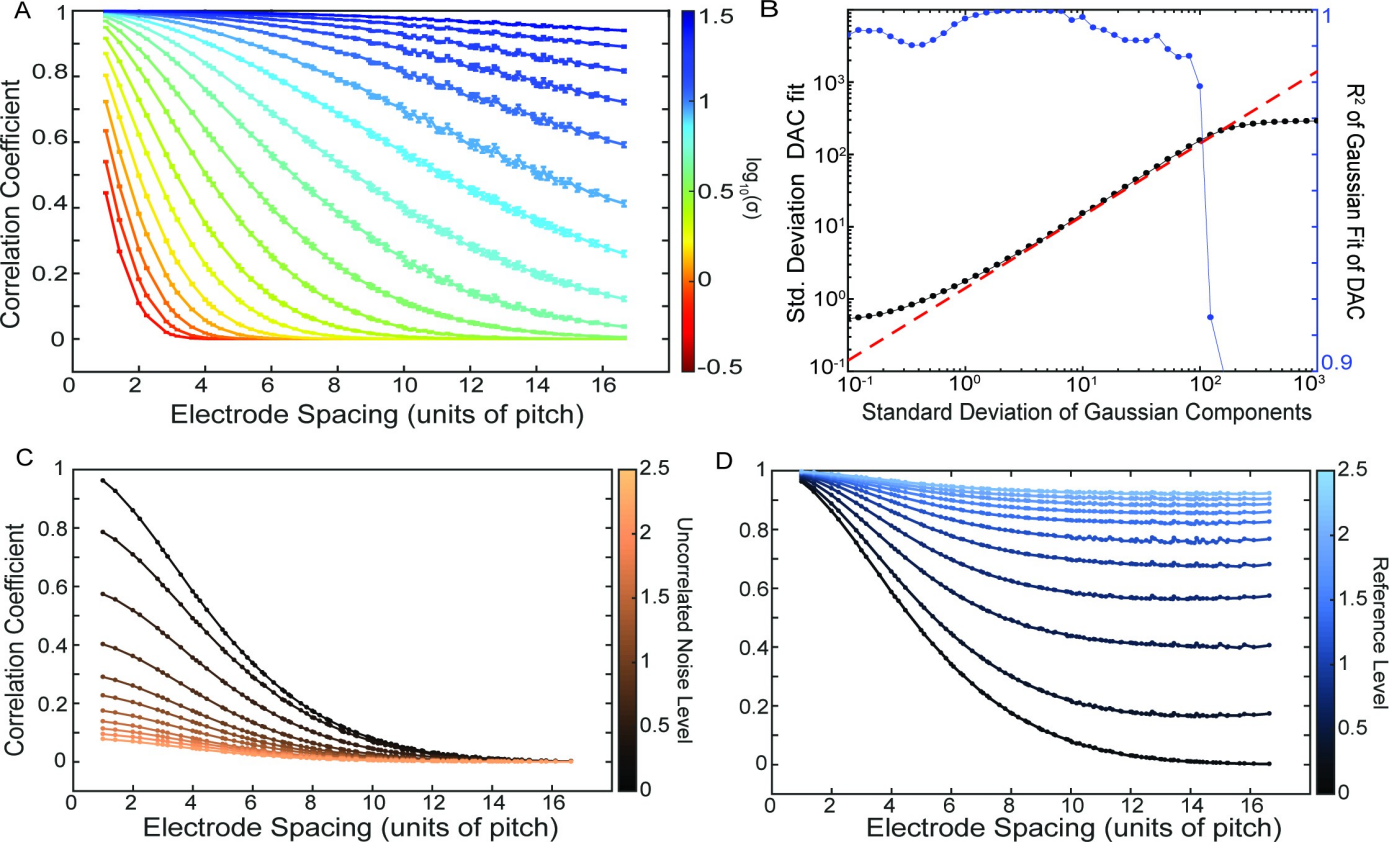
The effects of parameters of the Gaussian component modeling on the DAC curves. (A) The DAC curves for different Gaussian standard deviations averaged over 200 iterations of randomly generated components (in arbitrary units) (B) The results of Gaussian fits to the DAC from (A). The black dots are the standard deviation of the fit, and the red dashed line is the theoretical value of square root of 2 larger than the component standard deviation. The R^2^ values are plotted in blue to indicate where the fit is no longer appropriate. (C,D) The effect on the DAC of adding uncorrelated noise and a reference signal, respectively, to the Gaussian components.

To explore the connection between the ICA/PCA decomposition of the data into components and the correlation we start with a model how the spatial extent of the components affects the DAC. Component weights are modeled as two-dimensional Gaussian distributions sampled on a square grid of “electrodes”. The resulting correlation vs. distance curves are well-approximated by Gaussians, and we find a direct correlation between the width of the component Gaussians and the standard deviation parameter of the fit of the correlation vs. distance curve as shown in Fig 2. The relationship between the two is linear in the limit that there are many components that are sufficiently sampled by the grid of electrodes. The addition of uncorrelated noise to all channels decreases all correlation values by a factor, and the effect of having a reference signal added to every channel is to increase all correlation values. The effect of the noise is more apparent at small distances where even with the Gaussian components, the apparent *y*-intercept of the DAC drops as noise is added. On the other hand, the reference has the effect of raising the asymptotic value of the DAC for large distances.

We also directly connected the components to the DAC through the weight matrices (mixing matrices in ICA). Applying the ICA unmixing matrix (the inverse of the mixing matrix) to the data will decorrelate the data, and the unmixing matrix can be arbitrarily rescaled, therefore it is always possible for all of the components to have unit variance. This makes the ICA unmixing matrix a whitening, or sphering, matrix which is straightforwardly connected to the covariance matrix of the data because when multiplied by its transpose it must equal the covariance matrix. This allows a reduced, single-component covariance matrix to be calculated for each component of the mixing matrix separately, and the contribution of each component to the DAC can then be calculated.

### ICA decomposition of the recordings

**Fig 3:**
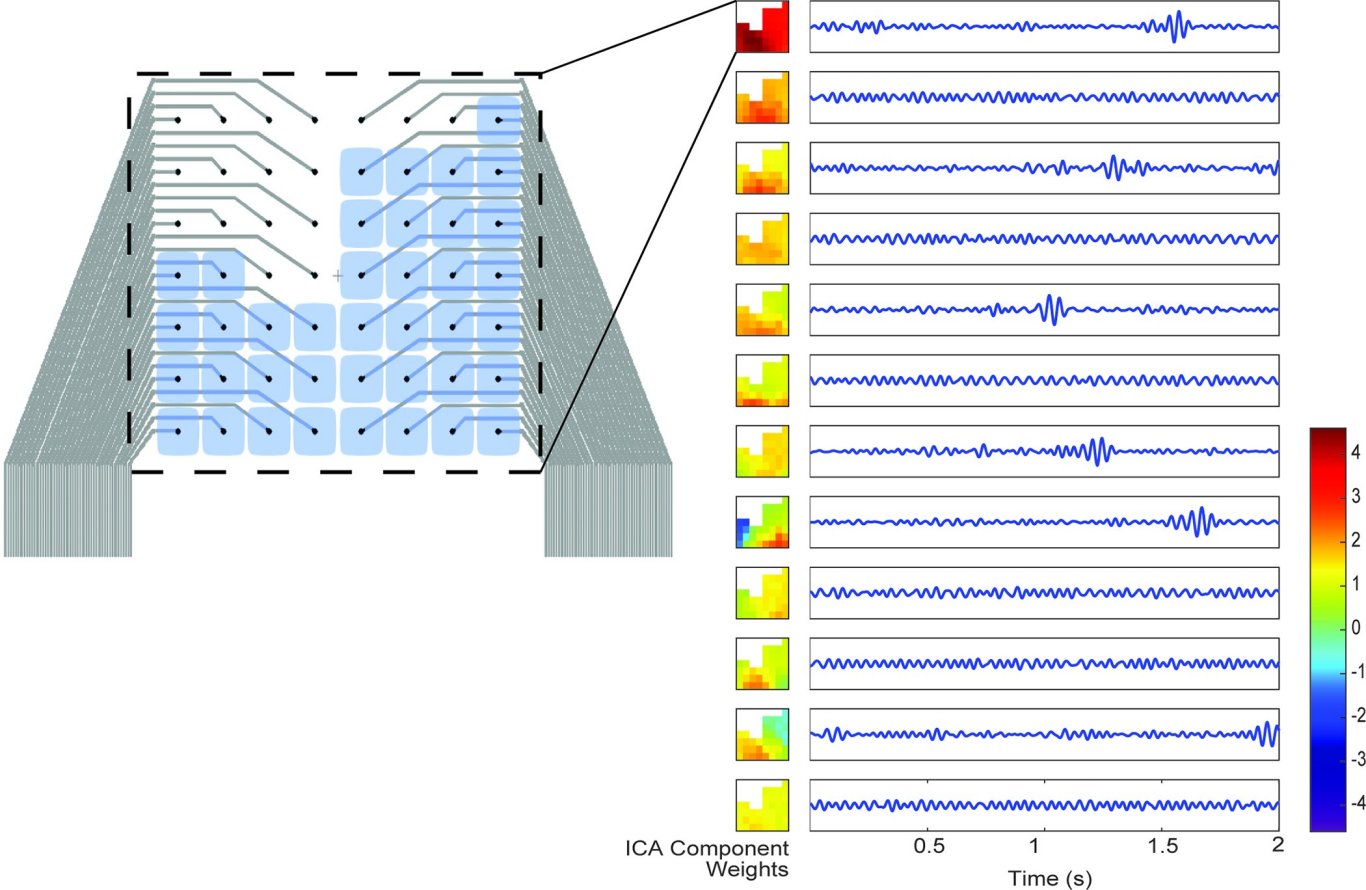
ICA components and their associated weights mapped to the grid geometry. On the left is an image of the electrode grid with the channels included in the analysis highlighted. An example of the output of ICA for a two second window from a human subject after a bandpass filter between 20 and 30 Hz. The left column is the weights associated with each component for the first 12 of 30 components plotted in the arrangement of the electrodes on the device. The right column are the components, or time series identified by ICA after normalizing to have variance of 1. The maps must be scaled inversely, and the overall amplitude of the component can be seen in the magnitude of the weights.

**Fig 4:**
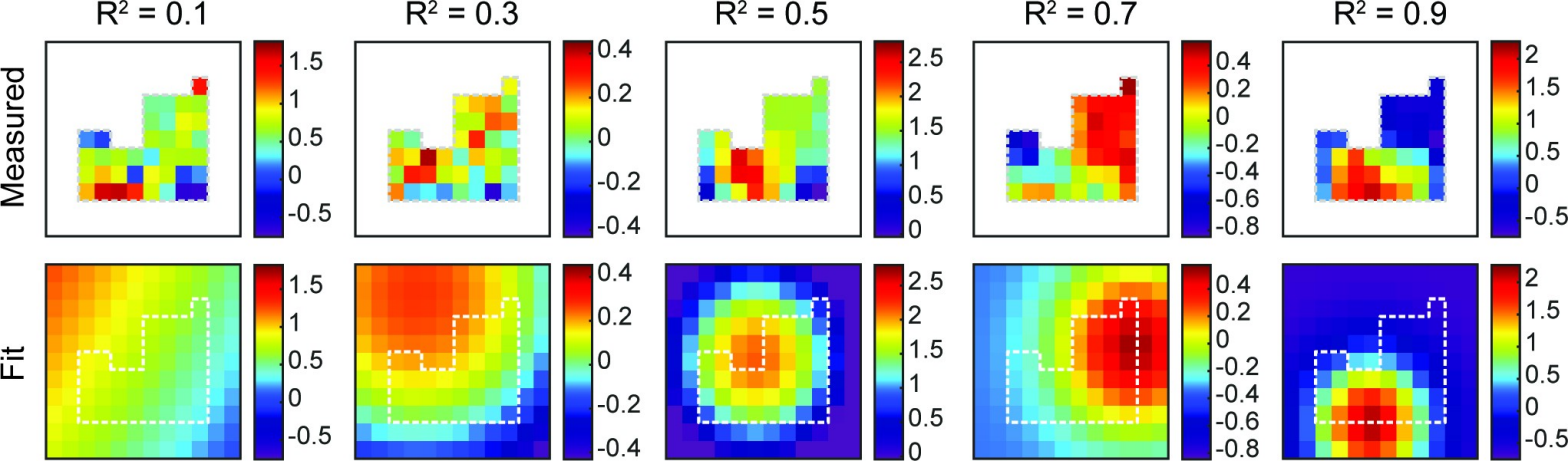
Example ICA components plotted on the electrode grid Examples of components with various R^2^ values (top), and their corresponding least-squares circular Gaussian fits (bottom).

**Fig 5:**
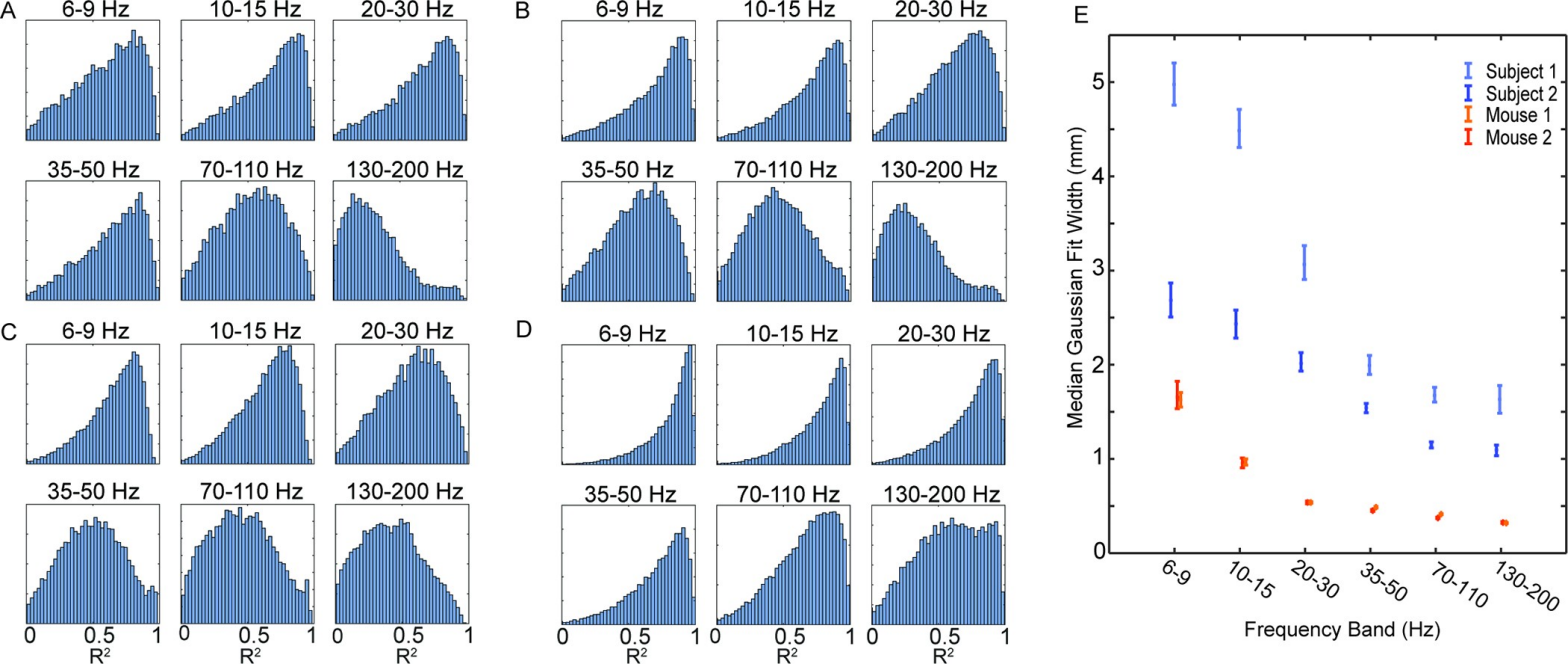
Histograms of the R^2^ values of fitting ICA components to a circular Gaussian function. The histograms are calculated separately for each subject and each frequency band across all 2.0 s windows. (A-B) R^2^ for two human subjects. (C-D) R^2^ for two mice. (E) The median width parameter of the Gaussian function for fits with R^2^ greater than 0.7 for all 4 subjects as a function of frequency band. The error bars are 95% confidence intervals obtained by a bootstrap analysis.

ICA is applied (using the EEGlab implementation, see Methods) to the same filtered 2 second windows as were used in the DAC calculation. The mixing matrices, when plotted in the arrangement of the electrodes, show readily apparent spatial patterns throughout the recordings as shown in Fig 3. As a way to quantify the spatial patterns in the component weights in the mixing matrices, we fit the weights as they are laid out on the brain to a circular two-dimensional Gaussian function. The goodness-of-fit gives a rough assessment of the smoothness of the mapped weights and their spatial gradients (see Fig 4). Of the parameters of the fit, the one with we expect to have the most relevance to the correlation is the standard deviation, or width, of the Gaussian. Larger widths would correspond to larger correlated areas, and as a result, a higher correlation at larger distances. This effect can be seen when comparing the median width values for each frequency band independently. As shown In Fig 5, the median Gaussian width decreases with frequency in agreement with the frequency dependence of the DAC, and that the components tend to be more Gaussian for lower frequencies. At the highest frequencies there is a marked decrease in the goodness of fit that may be due to lower signal-to-noise ratios expected as the 1/*f* decrease in the signal approaches the noise floor of the hardware.

**Fig 6:**
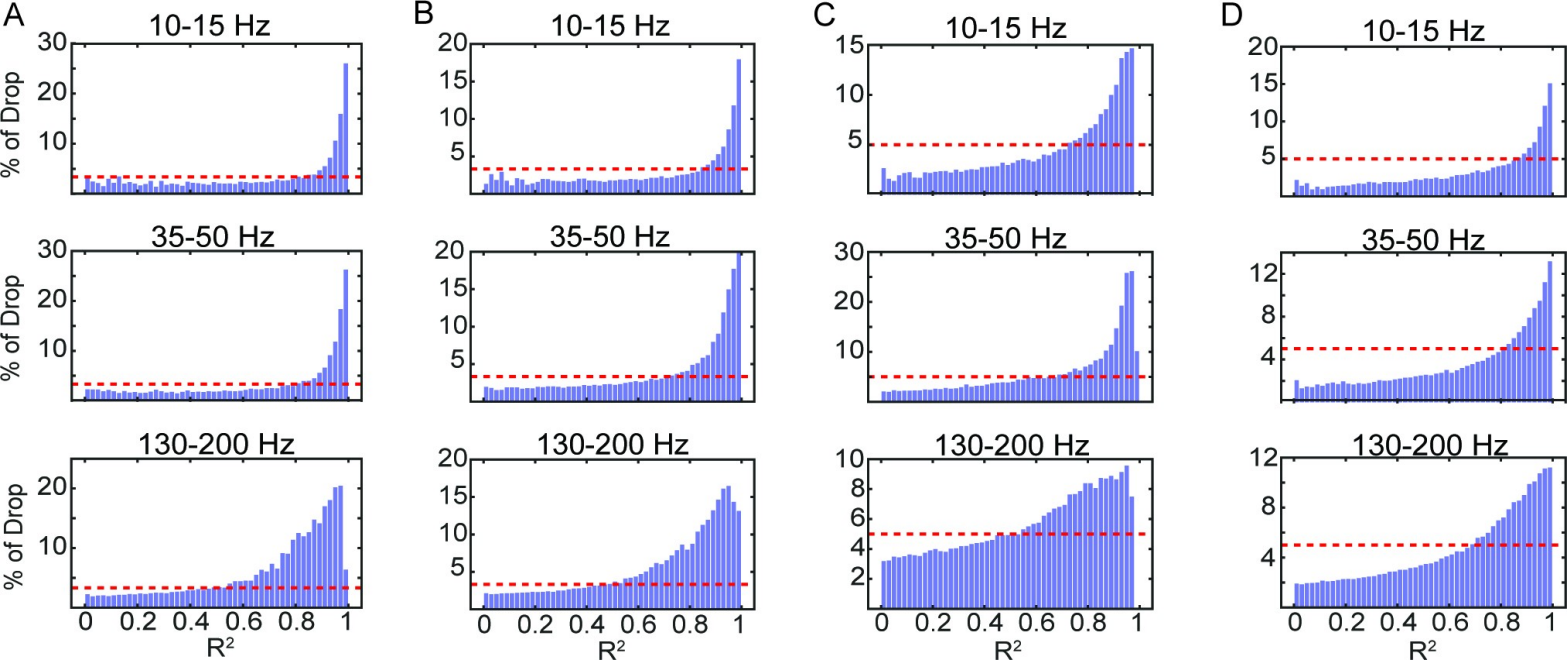
Contributions to the drop in the DAC as a function of goodness-of-fit. The percentage of the DAC drop explained by each component is averaged across components with similar (binned) R^2^ values. Therefore, each bar represents the average percentage of the DAC drop explained by components as a function of their values of R^2^, and are shown for 3 different frequencies and for each subject (A-D) as in Fig 4. The dashed red line represents the percentage that would be explained by each component if all components contributed equally.

By comparing the contributions of each ICA component to its Gaussian fit we find if and how the DAC curves are influenced by the spatial distribution of the component weights. The spatial distribution of the weights must explain the DAC curves, but to determine whether the Gaussian fits have any significance for the DAC in real data, the contribution of the components is plotted as a function of the R^2^ values of their fits in Fig 6. The higher R^2^ components account for a disproportionately large amount of the drop in the DAC with distance, and therefore the particular shape of the DAC is mostly attributable to the more Gaussian components. The correlation and variance are concentrated in more Gaussian components which shows that the larger, more significant components are generally roughly Gaussian (Supplement Fig 1).

As a control, PCA is substituted for ICA, and because it can also be rescaled into a whitening matrix, all of the analyses can be carried out in the same manner for PCA as for ICA. PCA is not a source separation algorithm like ICA, but in the case where there are sources that account for most of the variation these sources will show up in the first principal components. This can be seen in the R^2^ histograms (Supplement S2 Fig) in where the components are concentrated near 0, but there is also a smaller number of components very near 1.0 that account for much of the variance. These are the first few PCA components which are larger and more Gaussian. These more spatially Gaussian principal components account for much of the drop in correlation, so using PCA for comparison both shows the effectiveness of ICA in finding local sources, and that again, the more Gaussian components are more strongly tied to the drop in the DAC with distance.

The location of the reference electrode can have a large impact on the correlation values as shown in Fig 2D [31, 33–35]. The reference electrode subtracts the same signal from all of the recording channels and this will act to increase the correlation between any two channels if the reference is sufficiently uncorrelated with the signals. In mouse the reference electrode was placed subcutaneously and not on the skull, while in human the reference was multiple contacts within 10 cm of the of the micro-ECoG grid on the cortical surface. The latter are more likely to be active at the frequencies of interest, and even correlated with the unipolar signals measured at the grid. The reference electrode placement should always be taken into account when interpreting correlation or other measures of signal similarity, and that the relatively close reference used in the human subjects is not an ideal placement for studying DAC. Consequently, the DAC curves we obtained in Fig 1 should not be interpreted as the DAC corresponding to unipolar potentials (potentials measured against a theoretical reference potential of zero) which would be the ideal for studying spatial correlation across the brain. Using the methods in [36] we attempted to identify the reference signal in the human recordings, but a signal that matched the criteria was not found. Additionally, the reference will ideally be identified by ICA as a component with the same weight on every channel across the grid. In practice this is unlikely, but it may be identified in part and represented by components with relatively flat weights. In fact, this method was used in [37]. In our case it may be correlated with the unipolar surface potentials at the grid and could be mixed in with components of those.

A common method in ECoG to remove the true reference is to use the common average reference (CAR), at the expense of introducing a virtual reference which is also unknown. When ICA is applied after CAR, and fits are recalculated the distribution of R^2^ values is nearly unchanged (supplemental S4 Fig). While the mean Gaussian widths are significantly different after CAR (two-sample Kolmogorov-Smirnov test p > 0.05), they follow the unreferenced values closely but are slightly larger (19 +/- 9%). This shows that the references that were used did not have a large effect on the components and their spatial properties. The effect of CAR on the correlation is shown in supplemental S3 Fig, and the large difference between the original and CAR correlations can be understood through the effect of the CAR matrix discussed in the Methods. The amount subtracted from each component is uniform across all the channels and is equal to the average weight. Therefore, the shapes of the components are unchanged, but they are shifted such that they mean weight of each component across channels is zero. Reference effects are removed in this way due to their representation as a uniform component across all channels. This shifting of the weights can be seen in the data through the offset term of the Gaussian fit becoming strictly negative after CAR.

## Discussion

The results of our study indicate high degree of variability of the DAC, both within any set of data, as Fig 1 shows, and between datasets due to external factors such as where on the cortex the electrodes are placed. There is large variation across the 2.0 second windows as shown in Fig 1, that may reflect changes in ongoing activity. In fact, it has been shown that there are task-related changes in the DAC [13, 15, 38], however we did not find there to be task- or state-related changes in the DAC in the human recordings.

The particular curve of the DAC may change between time windows, recording epochs, subjects, and species, but a robust feature in our recordings and previous studies is the frequency dependence of the spatial correlation. This agrees with past studies that the responses in lower frequency bands are more spread out than in higher bands [13, 17, 26, 32, 39] and is evident in similar studies that used coherence instead of correlation, which is an inherently frequency dependent similarity metric. The coherence is plotted as a function of frequency, and in for ECoG data almost completely monotonically decreases with frequency [13–15].

The geometry of the ICA component weights offers a possible explanation of this frequency dependence. Two ways by which neighboring electrodes can be correlated are by volume conduction and coactivation of populations close to each electrode which produce distinct, but correlated potentials [40, 41]. In many cases there is a degree of both which contributes to the correlation, but for large distances where volume conduction is assumed to be negligible the presence of correlation is used as an indication of connectivity [28]. On the other hand, at the submillimeter scale we expect volume conduction may play a larger role. The presence of a single-peaked, radially symmetric, and smooth ICA weights map is consistent with volume conduction of the potentials, and the Gaussian fits show that many of the components fit this description. Coactivating regions could also be described by this shape but are not limited to it; there could be distinct, separated peaks, plateaus, or checkerboard-patterned regions.

The large and consistent difference in the DAC between human and mouse can also be explained by either larger coactivated areas of cortex or a larger effect of volume conduction in human cortex. The spread of a signal due to volume conduction in brain tissue would be the same in either species assuming they have similar conductivities, but human cortex has neurons with larger lateral spread of their dendritic and axonal trees and is roughly twice as thick as mouse cortex. The effect of volume conduction on deeper sources will spread the potentials they cause more widely across the cortical surface. Additionally, the size of functionally distinct cortical regions is larger in humans, and we are again left with the ambiguity between the two possible factors: the size of the correlated activity, and its spatial spread as in [26].

In our analysis the choice of ICA as the particular form of whitening and 2 dimensional Gaussians as the function used for fitting are not the only choices that could have been used, but they were chosen for simplicity and applicability to this analysis. As a fitting function, a 2-dimensional Gaussian was chosen for its simplicity and flexibility, and not due to any assumption that the components would take this particular form. The purpose of the fit is to identify smoothly varying component weights. The function is smooth on a small scale, with the only peak being the center, so that neighbor-to-neighbor oscillations in the weights will degrade the fit. It is also able to describe radially symmetric peaked distributions as well as flat linear gradients by being fit to a very wide Gaussian with its center far from the electrode grid. Alternatives more tailored to quantify the smoothness could be used such as taking the second spatial derivative of the component weights and finding smooth gradients and peaks by taking first spatial derivatives. These are harder to implement and interpret, and the high R^2^ values when using ICA and low ones from PCA show the fitting approach is able to describe the components while not being so flexible that it can be fit to any component weights.

Another reason for using the Gaussian fits is that they provide a single parameter that characterizes the size of the regions that contain the components. The mean width of the fits of each frequency band decreases with increasing frequency, and this may be due to the effect that frequency has on the spatial spread of LFP. Additionally, the fits provide another method of removing distant volume conducted activity similar to the ICA approach used in [37] by using both the center location of the Gaussian along with the width to identify components of the signals that are far from the grid location. This kind of ICA-based method as an alternative to standard re-referencing schemes has been proposed in [42].

On the other hand, the DAC curves are not fit to Gaussians for the data despite the modeling results that showed that Gaussian components have Gaussian DACs. We expect that using the same model, but with other peaked, but not necessarily radially symmetric distributions, will still result in monotonically decreasing DAC curves with a different shape. Additionally, the effect of noise and reference will add a predictable modification to the curve but adds additional unknown parameters. These may be estimated but this is confounded by the unknown effect of the actual non-Gaussian shape of the components, and the fit becomes more difficult to implement and interpret.

The component mixing matrix weights analysis requires any whitening matrix to separate the components, but ICA was chosen for this purpose. Commonly used whitening transformations are not intended to perform source separation, but ICA can be both a whitening and a blind source separation algorithm. PCA and factor analysis have been used for finding common sources in the data, but the assumptions about the data of ICA are more well suited to finding sources in electrophysiology, hence its popularity. Factor analysis is designed to find similar localized sources but is not easily modified to be a whitening transformation and its assumption of normally distributed components is incompatible with the sinusoidal nature of narrow bandpass filtered signals.

There are drawbacks to calculating ICA in separate frequency bands, as any broadband processes or ones that span frequency ranges between or across multiple bands will not be as accurately identified or be recognized as part of the same component. Still, ICA was calculated by frequency band so as to be calculated on the exact same windows and signals as the correlation, and due to the 1/*f* power spectrum typical of electrophysiology. The first consideration is necessary specifically for linking the correlation and the component mixing matrix through the covariance matrix, while the second is a general problem in applying ICA to LFP. ICA may less accurately separate sources whose power is concentrated in higher frequencies due to the much larger power present in lower frequencies biasing ICA towards identifying sources concentrated in those. In addition, if PCA is applied as a pre-processing step, the components that contain some high frequency sources may even be discarded.

ICA was applied only to small time windows in addition to narrow frequency bands. This has similar drawbacks in terms of the effectiveness of ICA because it limits the number of observations which ICA can use to identify source. For the same reason as before, consistency with the segments analyzed for correlation, the 2 second windows are needed. Also, this length of time may be appropriate because the components were found to vary even between adjacent windows. However, there is some consistency in the ICA mixing matrices across time—that is very similar components show up repeatedly, but not consistently. This suggests that the time scale of the duration of stable components may be 2 seconds or less, and perhaps ICA would be better suited to even shorter windows for this data in future work to avoid temporal fluctuations in source strengths which does not fit the assumption in ICA of time-invariant mixing matrices.

The curve generated by averaging the correlation over many contacts offers some guidance as to how the signals will be related for a given electrode spacing, but it is more straightforward to choose a spacing when given a measure of the spatial extent of the activity. The two are linked, and as has been shown previously with the correlation, the frequency has a strong effect on the spread of potentials measured at the surface of the brain. Electrodes spaced less than a millimeter apart are more suited to higher frequencies or to smaller animals than humans, but even with very limited cortical coverage volume conduction still allows activity that is not directly under the grid to be recorded.

## Methods

### Ethics statement

All human subject research was conducted in accordance with a study protocol that was reviewed and approved by the UC San Diego Health Institutional Review Board (protocol #121090). All animal work procedures were in accordance with a protocol approved by the Institutional Animal Care and Use Committees of UC San Diego (protocol S07360). Anesthetics used in mice were isoflurane and alpha-chloralose. Pentobarbital injection was used as the method of euthanasia.

### Human intraoperative recording

The details of the electrodes, their preparation, their implantation, and the recording setup are given in [18–20, 43]. Subjects who were undergoing awake craniotomy surgeries were chosen for recording. The entire section of hardware up to the amplifiers had to be sterilized due to its proximity to the surgical field. The electronics underwent STERRAD sterilization, and it was important to ensure that the devices would remain intact after autoclave sterilization [43]. The electrode grid was placed over STG, with larger electrodes within a few centimeters of the grid used as reference electrodes. The ground electrode was placed in the scalp. Recording was sampled at 20 kS/s, with a built-in high pass filter at 0.1 Hz and low pass filter at 7500 Hz.

### Mouse acute recording

ICR mice weighing 25–35 g were used in the experiments. The mice were placed on a heating pad and anesthetized with isoflurane. A femoral artery was catheterized for monitoring and injection, and a tracheotomy was performed for ventilation of the mice. After fixing the skull to a holder using dental acrylic, a craniotomy and durotomy were performed over the right whisker barrel and surrounding cortex. A well was formed around the craniotomy using dental acrylic, and the exposure was kept filled with artificial CSF until the electrode array was placed. Prior to recording the mice were administered pancuronium and artificially ventilated, and prior to stimulus trials the mice were switched to alpha-chloralose anesthesia. The exposure was dried prior to the electrode placement, and then covered with 0.7% agarose made with artificial CSF. The electrodes arrays used were arranged in square grids with either 0.2 or 0.25 mm spacing, and either 50 micron or variable diameter contact sizes. The reference electrode was silver-chloride ball placed between muscle tissue exposed for the craniotomy. Whisker flick stimuli were presented every 2 seconds, and recordings included both spontaneous epochs as well as periods with stimulation.

### Recording and preprocessing

All data was recorded with an Intan RHD2000 system, and the recordings were sampled at 20 kS/s with a high pass filter at 0.1 Hz and low pass filter at 7500 Hz. Channels that by visual inspection were highly contaminated with noise were assumed to be from damaged electrodes removed from further analysis.

Data was then downsampled to 4 kS/s, and 6 FIR bandpass filters were applied, chosen such that they span about 0.6 octaves, have no overlap, not include 60 Hz, and roughly correspond to physiological bands (theta, alpha, beta, gamma, high gamma): 6–9 Hz, 10–15 Hz, 20–30 Hz, 35–50 Hz, 70–110 Hz, and 130–200 Hz.

Windows of time where the reference signal was more than 35 uV from zero, any one channel was outside +/- 4 mV, or the signal in the highest band was more than 20 times the RMS in that band were marked as potential artifacts and excluded along with 750 ms prior and 1.25 s after. Regions that were not removed in this way but were shorter than 6 seconds were also excluded.

### Distance-averaged correlation

The data was segmented into continuous 2 s windows. For each window correlation was calculated using Pearson’s correlation coefficient for every possible pair of electrodes on the grid. Each channel pair also has an associated inter-electrode distance, and the correlation vs. distance plots are the result of averaging the pairwise correlations with all equally spaced pairs. For a subject the average correlation is calculated by pooling all correlations across time and averaging the values by distance.

### Component analysis and modeling

PCA and ICA decompose the data matrix, *s*, into linear combinations of components, *z*, with the transformation, mixing, or weight matrix, *W*,

s=Wz

The components are all uncorrelated with every other component. Therefore, the mixing matrix obtained from either ICA or PCA can be used to whiten the data—which is to linearly transform the data such that the covariance matrix of the transformed data is the identity matrix. PCA is commonly used as for whitening data, and for our purpose ICA can be defined such that it is a whitening transform due to the ambiguity in the scaling of the components. The components can be arbitrarily scaled so long as the weights are scaled inversely such that the original data is unchanged. We make use of this choice to conveniently express the covariance of the data as a function of the weights

$$\mathrm{cov}(s)=\mathrm{cov}(Wz)=W\mathrm{cov}(z)W^{T}=WIW^{T}=WW^{T}$$

Using this result, the correlation matrix can be computed directly from the mixing matrix. For the model we can start with mixing matrices with each component’s weights being drawn from a two-dimensional Gaussian

$$W_{i,j}=A_{j}e^{-\frac{{\left(x_{j}-x_{i}\right)}^{2}+{\left(y_{j}-y_{i}\right)}^{2}}{2\;\sigma_{j}^{2}}}$$

where *i* is the channel with position (*x*_*i*_, *y*_*i*_), and *j* is the component with location (*x*_*j*_, *y*_*j*_) and standard deviation 𝜎_j_. For our model we “sample” the components on a 10x15 square grid with 150 components, one per channel, whose center positions are drawn from a uniform random distribution on the 2D space covered by the grid plus one fifth of the standard deviation of the component for which the center is being determined on either side. The amplitudes of each component are drawn from a uniform random distribution between 0.5 and 1.5, sorted in descending order and then scaled by *e*^*-0*.*1 k*^, where *k* is 1,2,3… corresponding to the first, second, third, etc. amplitude. This is to mimic the trend of decreasing variance for components typically obtained from PCA and ICA. Once the mixing matrix is determined the covariance, *Σ*, is given as before by

$$\Sigma =WW^{T}$$

The properties of products of Gaussians allows the covariance elements to be rewritten as

$$\Sigma_{i,j}=\sum_{k=1}^{N}W_{i,k}W_{j,k}=\sum_{k=1}^{N}\;e^{-\frac{{\left(x_{k}-x_{i}\right)}^{2}+{\left(x_{k}-x_{j}\right)}^{2}+{\left(y_{k}-y_{i}\right)}^{2}+{\left(y_{k}-y_{j}\right)}^{2}}{2\sigma^{2}}}$$

$$=\sum_{k=1}^{N}\;e^{-\frac{2{\left(x_{k}-\frac{1}{2}(x_{i}+x_{j})\right)}^{2}}{2\sigma^{2}}}e^{-\frac{2{\left(y_{k}-\frac{1}{2}(y_{i}+y_{j})\right)}^{2}}{2\sigma^{2}}}e^{-\frac{\frac{1}{2}{\left(x_{i}-x_{j}\right)}^{2}+\frac{1}{2}{\left(y_{i}-y_{j}\right)}^{2}}{2\sigma^{2}}}$$

Separating the terms that involve the distance between channels *i* and *j* gives

$$\Sigma_{i,j}=e^{-\frac{d_{ij}^{2}}{2{\left(\sqrt{2}\sigma \right)}^{2}}}\sum_{k=1}^{N}e^{-\frac{2{\left(x_{k}-\frac{1}{2}(x_{i}+x_{j})\right)}^{2}}{2\sigma^{2}}}e^{-\frac{2{\left(y_{k}-\frac{1}{2}(y_{i}+y_{j})\right)}^{2}}{2\sigma^{2}}}=F_{i,j}e^{-\frac{d_{ij}^{2}}{2{\left(\sqrt{2}\sigma \right)}^{2}}}$$

Each element is the product of a Gaussian function of the distance between channels and *F*_*i*,*j*_, a sum over 2D Gaussian functions of the component positions centered at the average location of two electrode positions, which given a fixed set of component locations, is a function of the two electrodes’ positions. For uniformly distributed component positions *x*_*k*_, *F*_*i*,*j*_ looks like a discrete approximation of the integral over *x*_*k*_ of the 2D Gaussian. In the limit of an infinite number of components uniformly distributed across a sufficiently large area it becomes proportional to the integral

$$F_{i,j}\propto \int_{-\infty }^{\infty }\int_{-\infty }^{\infty }e^{-\frac{2{\left(x_{k}-\frac{1}{2}(x_{i}+x_{j})\right)}^{2}}{2\sigma^{2}}}e^{-\frac{2{\left(y_{k}-\frac{1}{2}(y_{i}+y_{j})\right)}^{2}}{2\sigma^{2}}}dx_{k}\;dy_{k}=F_{0}$$

and given that this integral is not a function of the center position of the Gaussian, in this limit *F* is a constant regardless of the positions of channels *i* and *j*. In this limit the correlation matrix is exactly a Gaussian function of the distance between the channel pairs with a standard deviation square-root of 2 larger than the standard deviation of the components that generated it:

$$r_{i,j}=\frac{F_{0}e^{-\frac{d_{ij}^{2}}{2{\left(\sqrt{2}\sigma \right)}^{2}}}}{\sqrt{\left(F_{0})(F_{0}\right)}}=e^{-\frac{d_{ij}^{2}}{2{\left(\sqrt{2}\sigma \right)}^{2}}}$$

For the above form of the correlation to be valid doesn’t require that *F* is near the limit of infinite components, rather that *F* is not a function of the electrode pair *i* and *j*. For the correlation to take the form above on average is an even weaker condition that *F* can be a weak function of the electrode pair in relation to the distance term so that the small factors multiplying the distance term will tend to cancel when averaged over equidistant pairs and multiple DAC’s.

To include the effect of noise specific to each channel, and uncorrelated from the activity or noise on the other channels, a new component has to be created for each noise element because every component is required to be uncorrelated to all other components. This results in a diagonal matrix of weights which we model as each having the same amplitude, *ϵ*, across channels

$$W_{noisei,j}=\epsilon \delta_{ij}$$

Where ẟ_ij_ is the Kronecker delta, not the distance used previously. The effect of a reference electrode which is added to all channels can be modeled as a single component with a constant weight vector across all channels

$$W_{ref}=\rho$$

so that the modified mixing matrix is the original mixing matrix with additional columns for the noise and reference

$$W^{\prime }=\left(\begin{array}{ccc} W & W_{noise} & W_{ref} \end{array}\right)=\left(\begin{array}{ccccc} & \epsilon & 0 & & \rho \\ W & 0 & \epsilon & & \rho \\ & & & \ddots & \vdots \end{array}\right)$$

The modified covariance matrix is now given by

$$\Sigma_{i,j}^{\prime }=\Sigma_{i,j}+\epsilon \delta_{ij}+\;\rho$$

and its modified correlation matrix *r’* is given by

$$r_{i,j}^{\prime }=\frac{\Sigma_{i,j}+\epsilon \delta_{ij}+\rho }{\sqrt{\left(\Sigma_{i,i}+\epsilon +\rho )(\Sigma_{j,j}+\epsilon +\rho \right)}}$$

### Independent component analysis

ICA was applied using the *runica()* function from EEGLab [44] to the same filtered and segmented 2 second windows that were used to compute the DAC. The built-in PCA option was used to apply PCA prior to ICA as a dimensionality reduction technique, and the ‘extended-ICA’ option was used. For human data with 56 channel grids the first 30 components were kept, and for mice with 32 channel grids the first 20 components were kept. In both cases the excluded components accounted for less than 5% of the variance in the data, and usually were close to 1%.

Component mixing matrices were fit using least squares fitting function lsqcruvefit() in MATLAB to a two-dimensional circular Gaussian function with 5 parameters

$$Ae^{\frac{{\left(x-B\right)}^{2}+{\left(y-C\right)}^{2}}{2D^{2}}}+E$$

with lower and upper bounds such that A be positive, B and C to force the center to lie within 40 grid pitches on either side, D to fit to Gaussians that have standard deviations larger than a single grid pitch.

The median width parameter D was chosen instead of the mean due to the distribution of values being skewed towards zero. In order to calculate the 95% confidence interval of the median a bootstrap with 5,000 resamples was used. Only widths corresponding to components with R^2^ over 0.7 to exclude components for which a Gaussian is not a good representation and the value of D may not be meaningful.

The individual contribution of each component to the overall covariance matrix can be found by re-calculating the covariance using only the desired component. Any entry in the covariance matrix is a sum over weights corresponding to all components.

$$\Sigma_{i,j}=\sum_{c=1}^{N}W_{i,c}W_{j,c}$$

Therefore, we define the reduced covariance corresponding to a single component, *c*, as just the terms involving that component. The sum of all of the reduced covariance matrices is therefore the full covariance matrix.

$$\Sigma_{i,j}^{c}=W_{i,c}W_{j,c}$$

The corresponding reduced correlation matrix cannot be calculated as usual

$$r_{i,j}=\frac{\Sigma_{i,j}}{\sqrt{\Sigma_{i,i}\Sigma_{j,j}}}$$

because all of the reduced correlation matrices would be identity matrices. Rather the reduced correlation is calculated using the reduced covariance in the numerator, and the full covariance in the denominator,

$$r_{i,j}^{c}=\frac{\Sigma_{i,j}^{c}}{\sqrt{\Sigma_{i,i}\Sigma_{j,j}}}$$

so that the sum of all components of the reduced correlation matrices is equal to the full correlation matrix.

The reduced correlations can be averaged by distance in the same manner as the full one. The contribution of each component to the variance, correlation, and drop in the correlation can be calculated using the reduced covariance and correlation. The variances of the channels are the diagonal entries of the covariance matrix, and we will define the overall variance (across channels) as the trace of the covariance matrix. The variance across channels for a given component is then given by

$$Var^{c}=\sum_{i=1}^{N}W_{i,c}W_{i,c}$$

such that the overall variance is the sum over components, and the percentage of the variance explained by each component is the component variance divided by the overall variance.

We also want to know the contribution of each component to the DAC. The contribution to the DAC is a similar measure to the contribution to the variance, but the contribution to the drop in the DAC is more relevant for explaining the shape of the correlation vs. distance curve. To calculate these, the DAC curve for each reduced correlation matrix is calculated in the same manner as for correlation matrices. The drop in the DAC due to each component is calculated by subtracting the zero-distance value of the DAC from all the values, and it retains the desired property that the sum over all components is equal to the drop in the full DAC.

To summarize the amount each component contributes to the drop in the DAC into a single quantity, the percentage explained is calculated at each distance, and then averaged over all distances. With this, each component can be assigned a percentage of the total variance, total DAC, and total drop in the DAC.

### Common average reference

The common average reference can be computed with the matrix

$$\frac{1}{N}J$$

Where *N* is the number of electrodes and *J* is the *NxN* matrix of ones. The product of this matrix with *s* computes the average signal and is subtracted from the original signal to yield the CAR version of the signal

$$s_{CAR}=s-\bar{s}=s-\frac{1}{N}Js=\left[I-\frac{1}{N}J\right]s=Cs$$

The covariance of the signals after CAR is then

$$\mathrm{cov}(s_{CAR})=C\;\mathrm{cov}(s)C^{T}$$

The effect on the component-based representation is

$$s_{CAR}=Cs=CWz$$

It is important to note that this modification of the weight matrix applies to the weight matrix obtained without the change of reference. When CAR is applied to the data the temporal components identified by a whitening algorithm such as PCA are not necessarily the same.

## Supporting information

S1 Fig Histograms of variance and correlation explained by goodness-of-fit. To show how the values of R^2^, amount of variance, and drop in the DAC are distributed they are plotted as histograms across R^2^ bins. In blue is the same information as in Fig 3 with larger bins. In green the percentage of the variance explained, and in yellow the percentage of the drop in the correlation. (A) Subject Subject 2 and (B) mouse mouse 2.
(TIF)

S2 Fig PCA histograms of variance and correlation explained by goodness-of-fit. The same as S1 Fig when PCA is used instead of ICA for (A) subject 2 and (B) mouse 1.
(TIF)

S3 Fig DAC Results for other subjects and after CAR. The DAC for the 2 cases not shown in Fig 1 are shown, and the CAR DAC results for all 4 subjects.
(TIF)

S4 Fig Comparison of components before and after CAR. Results for CAR are shown as in Fig 5 with the CAR in dark overlaid over the results of the data without re-referencing in gray.
(TIF)
