# Supplementary figures and images for: Correlation Structure in Micro-ECoG Recordings is Described by Spatially Coherent Components
Source: PLoS Comput Biol. 2019 Feb 11;15(2):e1006769. doi: 10.1371/journal.pcbi.1006769 (PMC6386410; doi:10.1371/journal.pcbi.1006769)

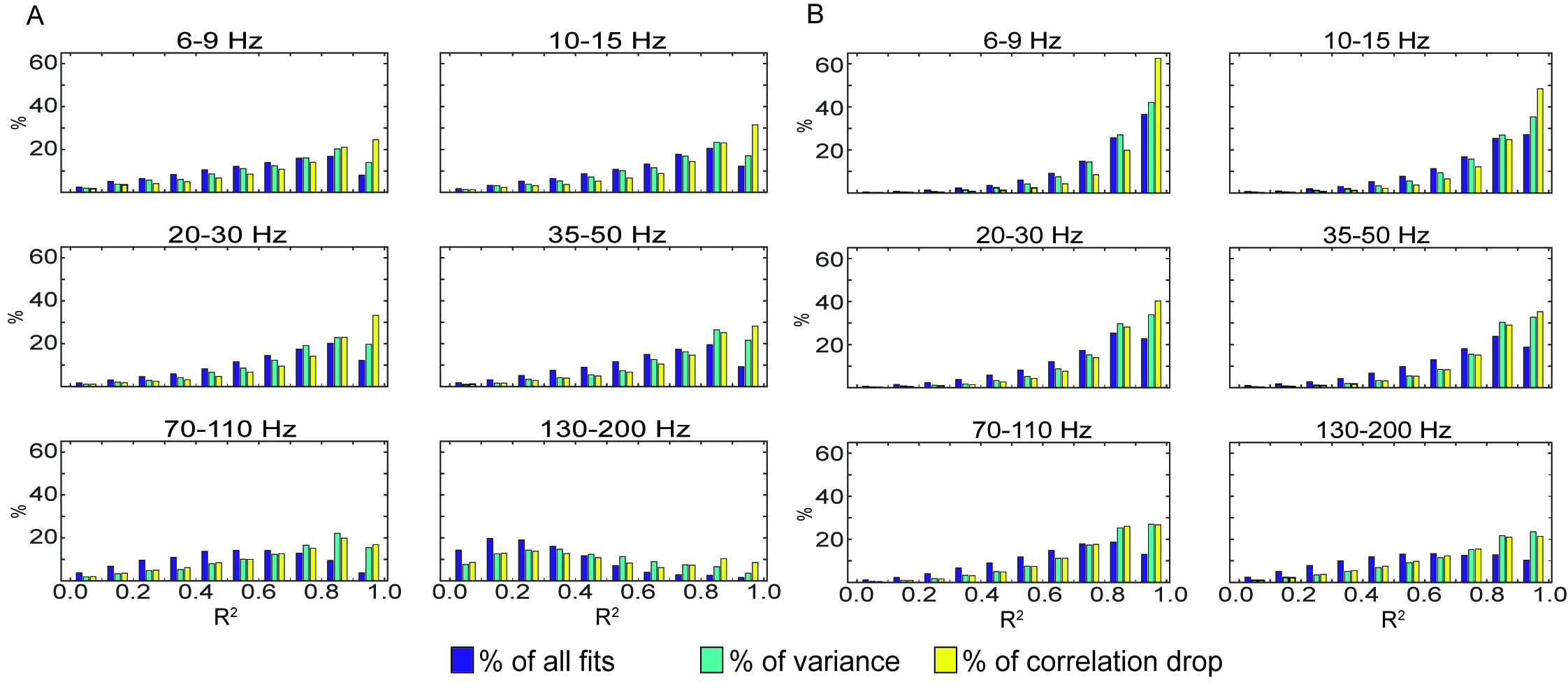

Supplement: S1 Fig — To show how the values of R2, amount of variance, and drop in the DAC are distributed they are plotted as histograms across R2 bins. In blue is the same information as in Fig 3 with larger bins. In green the percentage of the variance explained, and in yellow the percentage of the drop in the correlation. (A) Subject Subject 2 and (B) mouse mouse 2. (TIF) [file pcbi.1006769.s001.tif]

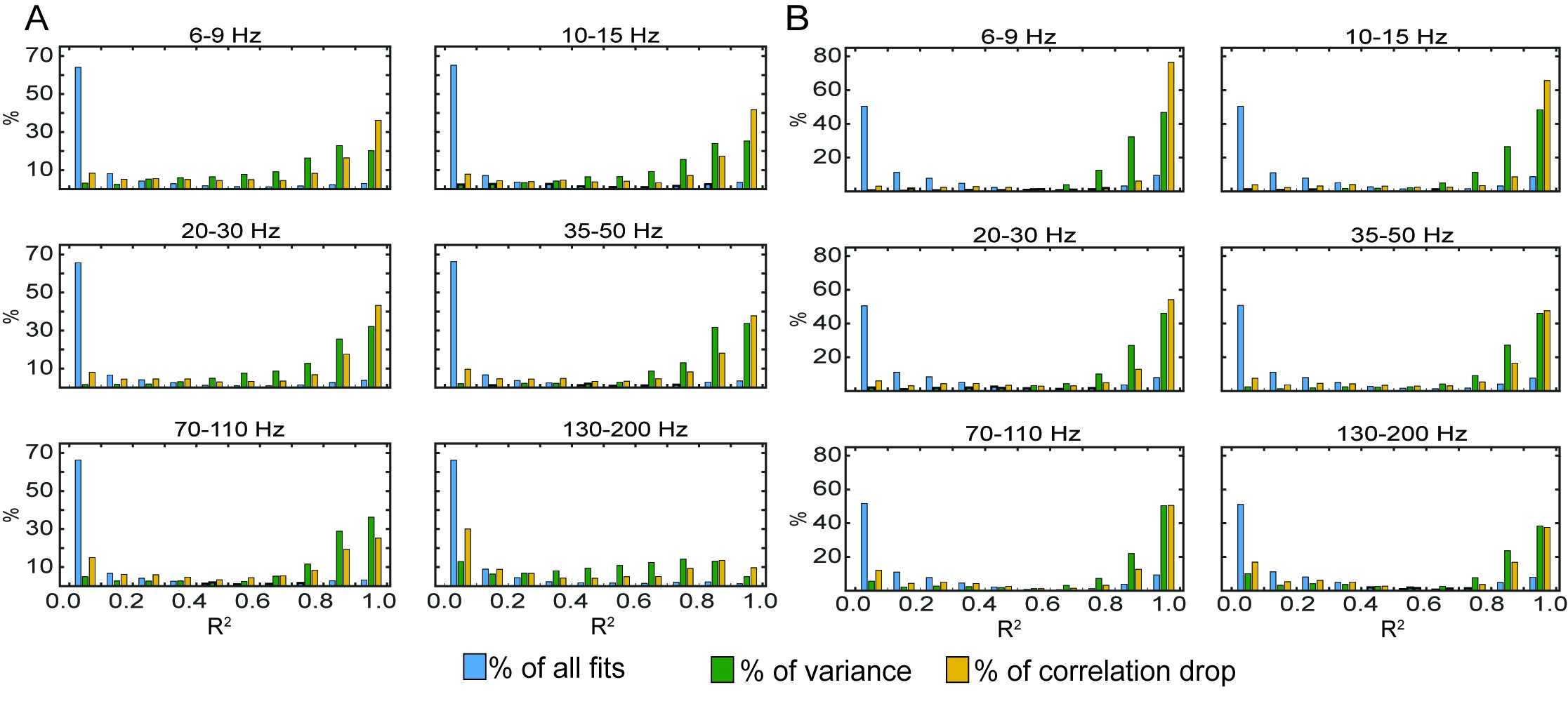

Supplement: S2 Fig — The same as S1 Fig when PCA is used instead of ICA for (A) subject 2 and (B) mouse 1. (TIF) [file pcbi.1006769.s002.tif]

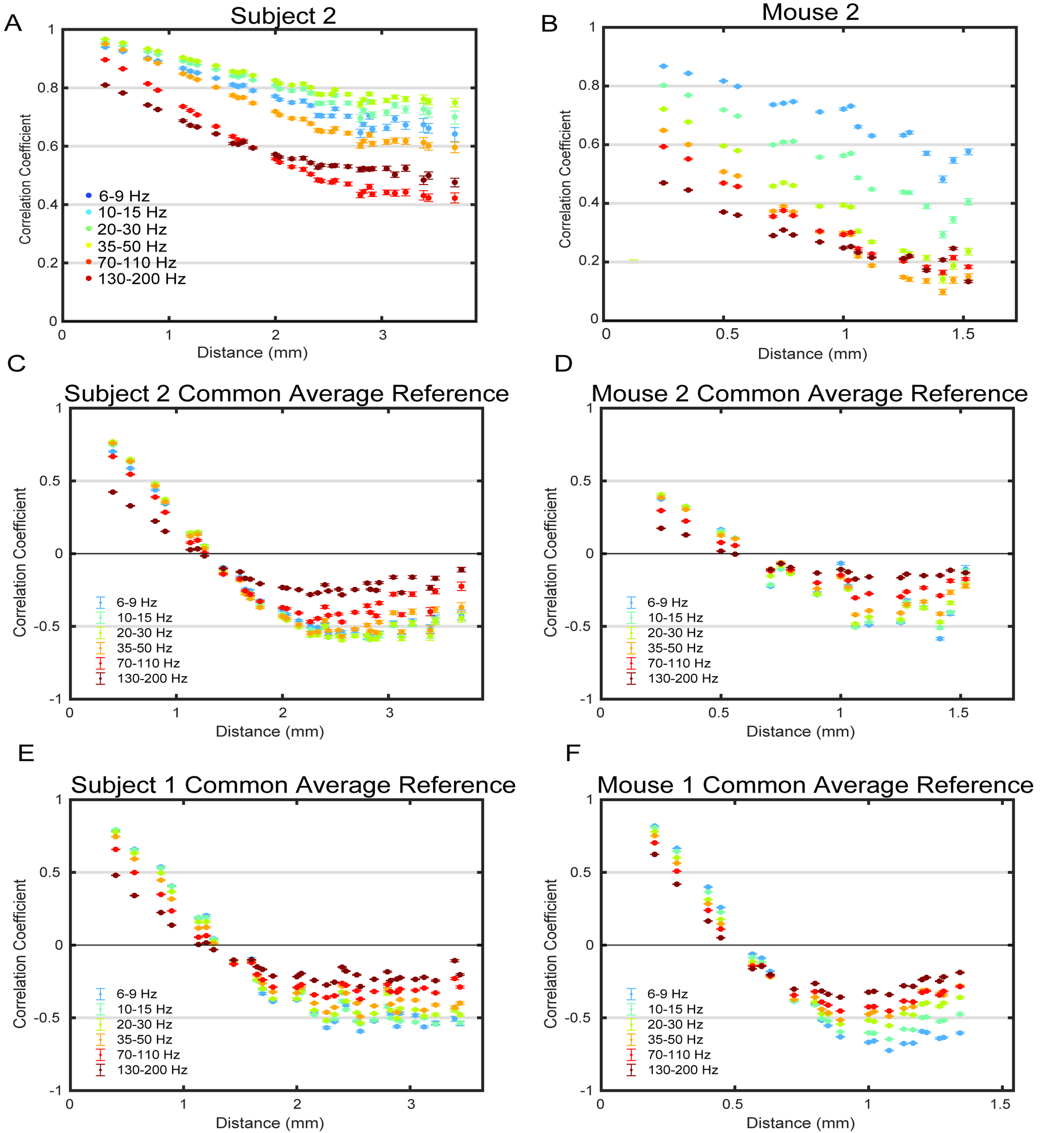

Supplement: S3 Fig — The DAC for the 2 cases not shown in Fig 1 are shown, and the CAR DAC results for all 4 subjects. (TIF) [file pcbi.1006769.s003.tif]

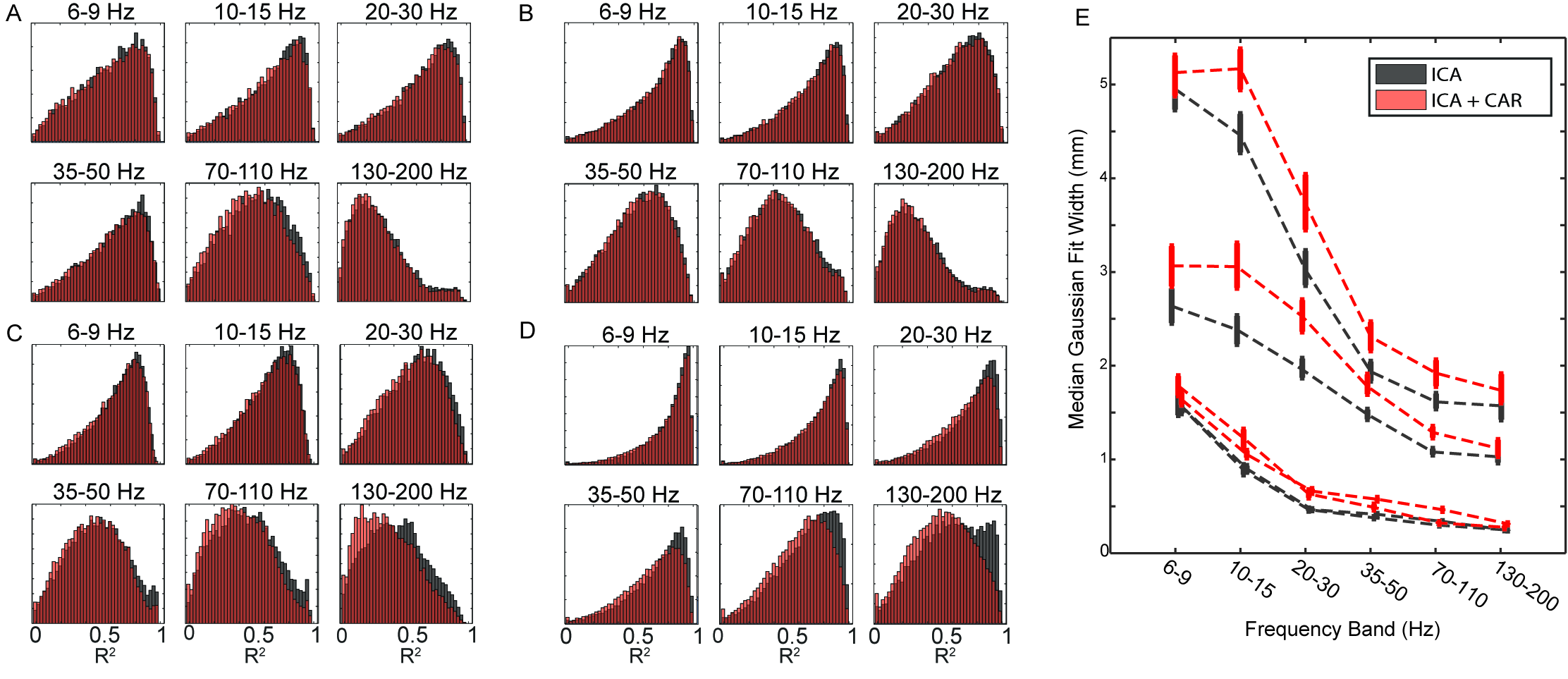

Supplement: S4 Fig — Results for CAR are shown as in Fig 5 with the CAR in dark overlaid over the results of the data without re-referencing in gray. (TIF) [file pcbi.1006769.s004.tif]
